# Comprehensive analysis of antimicrobial resistance in the Southwest Indian Ocean: focus on WHO critical and high priority pathogens

**DOI:** 10.3389/fpubh.2024.1357345

**Published:** 2024-04-02

**Authors:** Axel O. G. Hoarau, Patrick Mavingui, Guillaume Miltgen

**Affiliations:** ^1^Université de La Réunion, Unité Mixte de Recherche Processus Infectieux en Milieu Insulaire Tropical (UMR PIMIT), INSERM 1187, CNRS 9192, IRD 249, Sainte-Clotilde, La Réunion, France; ^2^Laboratoire de Bactériologie, CHU Félix Guyon, Saint-Denis, La Réunion, France; ^3^Centre Régional en Antibiothérapie (CRAtb) de La Réunion, Saint-Pierre, La Réunion, France

**Keywords:** antimicrobial resistance, Enterobacterales, *Pseudomonas* spp., *Acinetobacter* spp., *Enterococcus* spp., carbapenem resistance, vancomycin resistance, Indian Ocean

## Abstract

The spread of antimicrobial resistance (AMR) is a major global concern, and the islands of the Southwest Indian Ocean (SWIO) are not exempt from this phenomenon. As strategic crossroads between Southern Africa and the Indian subcontinent, these islands are constantly threatened by the importation of multidrug-resistant bacteria from these regions. In this systematic review, our aim was to assess the epidemiological situation of AMR in humans in the SWIO islands, focusing on bacterial species listed as priority by the World Health Organization. Specifically, we examined Enterobacterales, *Acinetobacter* spp., *Pseudomonas* spp. resistant to carbapenems, and *Enterococcus* spp. resistant to vancomycin. Our main objectives were to map the distribution of these resistant bacteria in the SWIO islands and identify the genes involved in their resistance mechanisms. We conducted literature review focusing on Comoros, Madagascar, Maldives, Mauritius, Mayotte, Reunion Island, Seychelles, Sri Lanka, and Zanzibar. Our findings revealed a growing interest in the investigation of these pathogens and provided evidence of their active circulation in many of the territories investigated. However, we also identified disparities in terms of data availability between the targeted bacteria and among the different territories, emphasizing the need to strengthen collaborative efforts to establish an efficient regional surveillance network.

## 1 Introduction

The spread of antimicrobial resistance (AMR) is recognized as an increasing global threat. It was estimated that in 2019, there were 4.95 million deaths worldwide associated with AMR, among which 1.27 million were directly attributable to AMR (1). This alarming situation originates from the emergence of multidrug-resistant strains and the lack of new effective therapeutic approaches. In 2017, the World Health Organization (WHO) established its first-ever priority list of antibiotic resistant pathogens (2). For instance, Gram-negative bacteria including Enterobacterales, *Acinetobacter* spp., and *Pseudomonas* spp. resistant to carbapenems, were classified as critical priority pathogens (2). Similarly, Gram-positive bacteria, such as *Enterococcus* spp. (specifically *Enterococcus faecium*), resistant to vancomycin (known as vancomycin resistant Enterococci or VRE), were classified as high priority pathogen (2).

The Southwest Indian Ocean (SWIO) is made up of multitude islands. Despite their relative isolation, these territories face significant pressure from the importation of antibiotic-resistant pathogens from Southern Africa and the Indian subcontinent, and they are highly concerned about AMR. In 2015, the Indian Ocean Commission (IOC), which includes Comoros, Madagascar, Mauritius, Reunion Island, and Seychelles, declared AMR a priority health issue (3). Gay et al. (4) conducted a systematic review of the literature in 2016 to assess the prevalence of AMR for bacterial species prone to develop multidrug resistance, and fecal-oral foodborne bacteria in humans and animals within the IOC and Mayotte. They pointed out that many resistant strains were circulating in both humans and animals (4). The main concerns were extended-spectrum β-lactamase-producing Enterobacterales and carbapenemase-producing Enterobacterales (CPE) (4).

In the present review, our aim is to portray the current AMR epidemiological situation in the SWIO, six years after the initial review. We will focus specifically on bacterial species that are registered on the WHO priority list, including Enterobacterales, *Acinetobacter* spp., and *Pseudomonas* spp. resistant to carbapenems, as well as *Enterococcus* spp. resistant to vancomycin. The objectives of our study were to *(i)* map the distribution of these resistant bacteria in the SWIO, and *(ii)* identify the specific resistance genes that may be involved.

## 2 Method

Our study was conducted between August and November 2023. We chose to include the following territories in the screening: Comoros, Madagascar, Maldives, Mauritius, Mayotte, Reunion Island, Seychelles, Sri Lanka, and Zanzibar. Following the Preferred Reporting Items for Systematic Reviews and Meta-Analyses (PRISMA) guidelines (5), we used published data by searching in the Google Scholar (RRID:SCR_008878), PubMed (RRID:SCR_004846), and Web of Science (RRID:SCR_022706) databases for articles, posters, and conference abstracts, in French or English from 1990 until November 2023. We collected relevant information on carbapenem-resistant Enterobacterales (previously named Enterobacteriaceae), *Pseudomonas* spp. and *Acinetobacter* spp. in each territory by combining bacteria names and locations with the keywords “carbapenem resistance” or “carbapenemase resistance.” Similarly, we collected information on vancomycin-resistant *Enterococcus* spp. by using the keywords “vancomycin resistance *Enterococcus”* or “vancomycin resistance Enterococci.” Only studies reporting the detection of at least one resistant isolate were included. References and data were discarded when original sources were not identified.

## 3 Results

**Figure 1:**
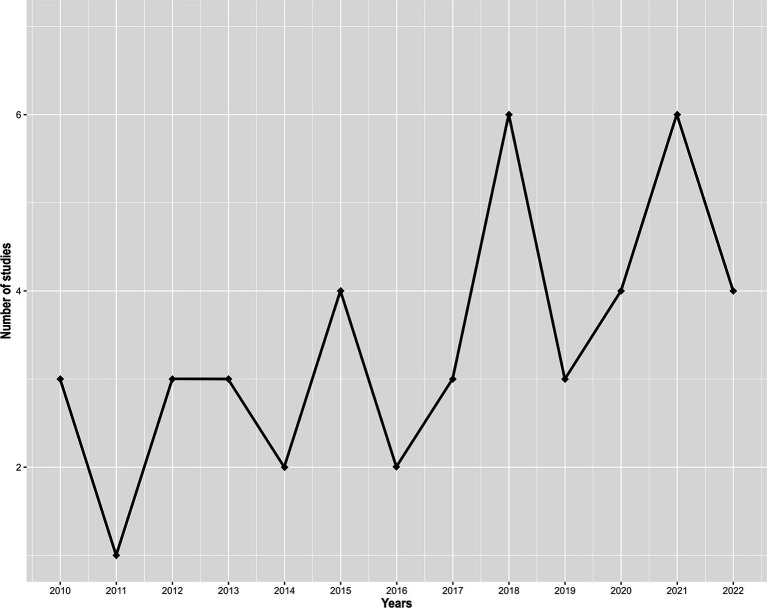
Number of studies identifying critical and high priority antibiotic-resistant bacteria in the Southwest Indian Ocean, per year, from 2010 to November 2023.

**Figure 2:**
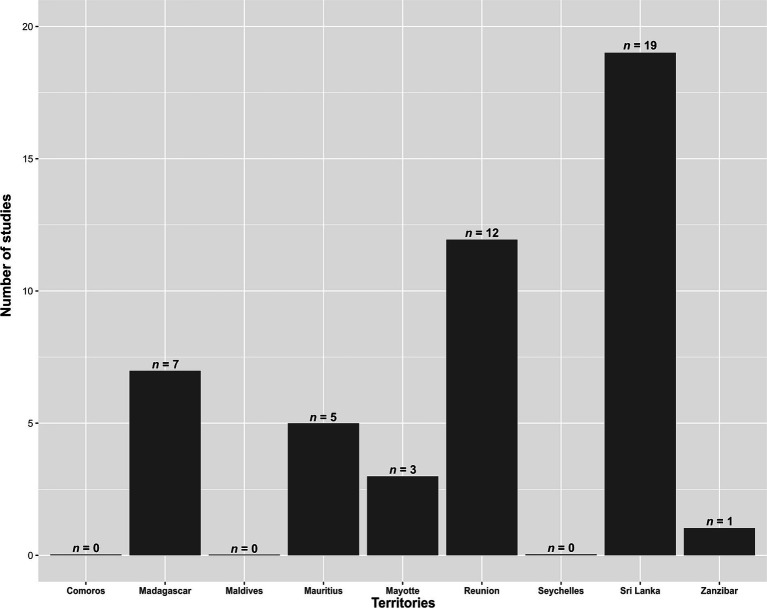
Number of studies identifying critical and high priority antibiotic-resistant bacteria in the Southwest Indian Ocean, for each investigated territory, from 2010 to November 2023.

A total of 102 studies were identified. Out of these, 58 were excluded for not meeting the inclusion criteria. The final analysis included 44 studies (Supplementary Table S1). None of these studies were published before 2010, and the number of studies has been increasing over time (Figure 1). The number of published studies varied according to territories (Figure 2), with Sri Lanka and Reunion Island accounting for 31 out of 44 studies (70.5%). Among the selected studies, 29 (65.9%) investigated the presence of antimicrobial resistance genes in addition to antimicrobial susceptibility studies (Supplementary Table S2). However, studies investigating the localization of these genes were scarce, representing only 27.2% (12/44) of the total studies (Supplementary Table S2). Carbapenem-resistant Gram-negative bacteria were reported in 42 out of 44 studies (95.5%), while VRE were reported in 13.6% (6/44) of the studies (Supplementary Table S2).

### 3.1 Enterobacterales resistant to carbapenems

**Figure 3:**
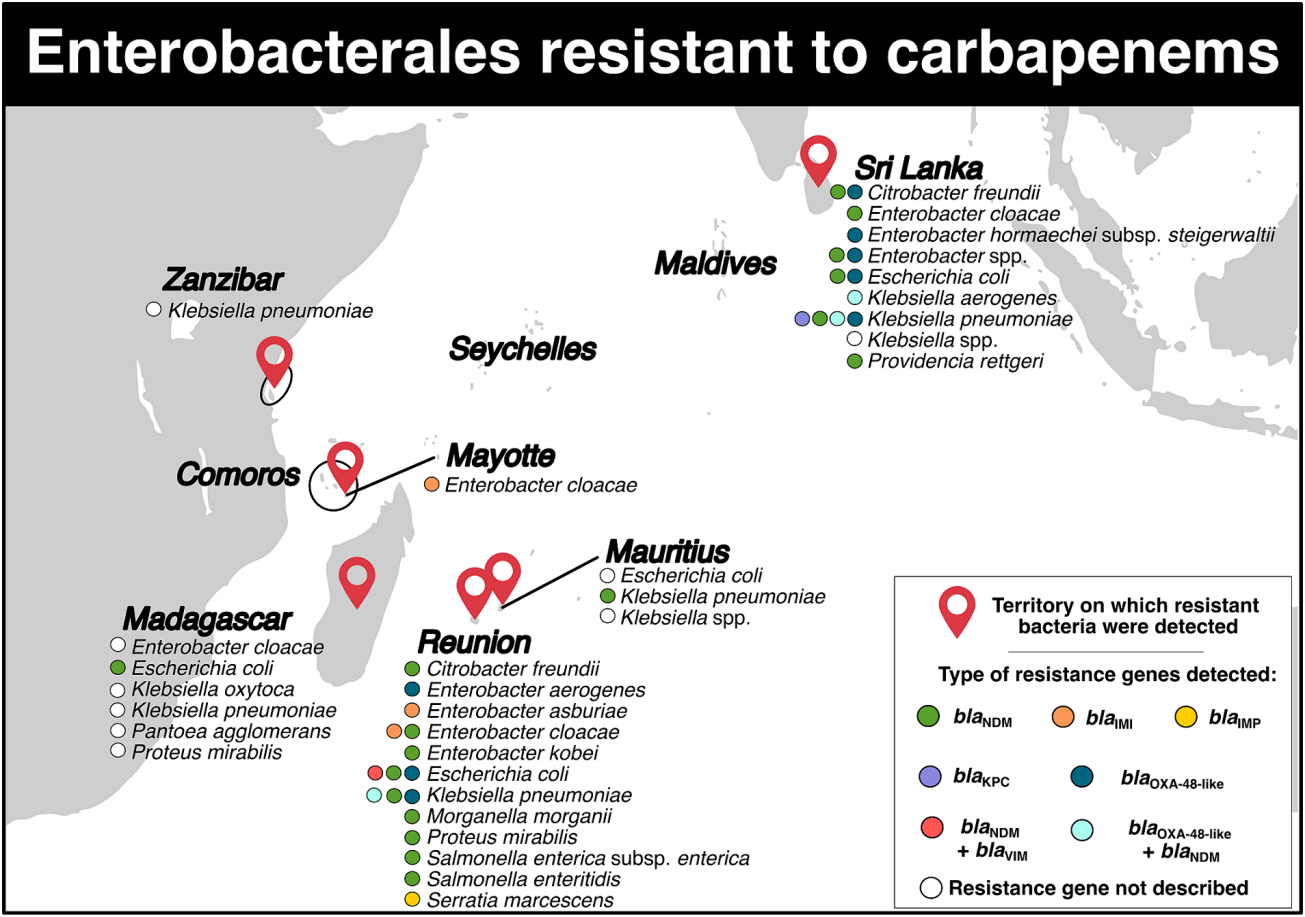
Distribution of carbapenem-resistant Enterobacterales and genes associated with resistance mechanisms in the Southwest Indian Ocean Islands.

Carbapenem-resistant Enterobacterales (CRE) were reported in six territories (Figure 3; Supplementary Tables S2, S3). In Madagascar, there were four studies available. A study conducted from 2011 to 2013, spanning three years, identified three *Enterobacter cloacae* isolates (resistance rate: 15.0%, 3/20), two *Escherichia coli* isolates (resistance rate: 2.3%, 2/89), six *Klebsiella pneumoniae* isolates (resistance rate: 17.1%, 6/35), three *Klebsiella oxytoca* isolates (resistance rate: 13.6%, 3/22), and one *Proteus mirabilis* isolate (resistance rate: 4.2%, 1/24) with carbapenem resistance in the community population (6). Another study reported one carbapenem-resistant *Pantoea agglomerans* isolate in a hospitalized patient (resistance rate: 11.1%,1/9) (7). Between 2015 and 2017, four patients (CRE carrier rate: 1.2%) presented with CRE (8). Between 2018 and 2019, a pregnant woman was found to have one carbapenem-resistant *E. coli* isolate (resistance rate: 0.6%, 1/168) carrying the *bla*_NDM-5_ gene (9).

For Mauritius, five studies were retained (Figure 3; Supplementary Table S2). In 2009, a carbapenemase-producing *K. pneumoniae* sequence type (ST) 231 isolate carrying the *bla*_NDM-1_ gene on a plasmid of IncA/C type was identified (10). In 2010, another carbapenem-resistant *Klebsiella* spp. isolate was discovered (11). In July 2014, a study reported resistant rates of 3.0% for *E. coli* and 9.0% for *Klebsiella* spp. (12). From 2015 to 2016, 23 CRE strains (resistance rate: 30.3%, 23/76) were isolated in an intensive care unit (13). Lastly, between 2015 and 2017, a total of nine patients tested positive for CRE (CRE carriers rate: 8.1%) (8).

On Mayotte Island, only two studies have reported CRE (Figure 3; Supplementary Table S2). A three-year study conducted between 2015 and 2017 identified 14 patients carrying CRE (CRE carriers rate: 0.9%) (8). Another study conducted over 16 months between 2015 and 2017 reported the presence of 18 isolates of *E. cloacae* ST820 that harbored the *bla*_IMI-1_ gene, which was carried on the integrative mobile element *Eclo*IMEX-8 (14).

In Figure 3 and Supplementary Table S2, we included seven studies related to Reunion Island. In November 2011 and March 2012, two patients who were repatriated from Mauritius and India were found to have *K. pneumoniae* and *Salmonella enterica* subsp. *enterica* serotype Westhampton carrying the *bla*_NDM-1_ gene, respectively (15). A retrospective observational multicenter study conducted between January 2010 and December 2015 reported several species of CRE in 36 patients (16). These species included *K. pneumoniae* (*bla*_NDM-1_), *E. coli* (*bla*_NDM-1_, *bla*_NDM-4_, *bla*_NDM-5_, *bla*_NDM-6_, *bla*_OXA-48_), *E. cloacae* (*bla*_NDM-1_ and *bla*_IMI-1_), *Citrobacter freundii* (*bla*_NDM-1_), *Morganella morganii* (*bla*_NDM-1_), *Enterobacter aerogenes* (*bla*_OXA-48_), *P. mirabilis* (*bla*_NDM-1_), and *Salmonella enteritidis* (*bla*_NDM-1_) (16). In early 2017, a patient was reported to be infected with carbapenemase-producing *K. pneumoniae*. This isolate harbored the *bla*_NDM-1-*like*_ gene. Additionally, an extensively-drug resistant *E. coli* carrying *bla*_NDM-1-*like*_ and *mcr-1* genes was also isolated from this patient (17). In the same year, a patient returning from Mauritius was found to be carrying a carbapenemase-producing *K. pneumoniae* isolate with the *bla*_NDM-1_ and *bla*_OXA-181_ genes (18). From 2011 to 2016, 61 Enterobacterales from 53 patients on Reunion Island were identified to have carbapenem resistance (19). Among them, 13 *E. coli* belonging to eight STs (ST10, ST12, ST167, ST349, ST354, ST405, ST410, ST1284) were recovered. These isolates carried five resistance genes: *bla*_NDM-1_, *bla*_NDM-4_, *bla*_NDM-5_, *bla*_NDM-6_, *bla*_OXA-181_, and one isolate even carried both *bla*_NDM-1_ and *bla*_VIM-2_ genes (19). Twenty-six carbapenemase-producing *K. pneumoniae* isolates belonging to 13 STs: ST14, ST15, ST17, ST37, ST101, ST147, ST307, ST359, ST524, ST1562, ST1864, ST2193, and ST4507 were also retrieved. They were found to carry *bla*_NDM-1_, *bla*_NDM-5_, *bla*_NDM-7_, and one isolate even carried both *bla*_NDM-1_ and *bla*_OXA-181_ (19). Nine *E. cloacae* isolates belonging to ST106, ST820, ST1304 and carrying *bla*_NDM-1_ and *bla*_IMI-1_ genes, six *C. freundii* isolates belonging to ST22, ST116, ST124, ST248, ST502 carrying *bla*_NDM-1_, three *Serratia marcescens* isolates carrying *bla*_IMP-10_, one *Enterobacter asburiae* isolate carrying *bla*_IMI-13_, one *Enterobacter kobei* isolate carrying *bla*_NDM-1_, one isolate of *P. mirabilis* and one isolate of *S. enterica* subsp. *enterica* harboring *bla*_NDM-1_ were detected (19). Between 2015 and 2017, a total of 10 patients tested positive for CRE (CRE carrier ratio: 0,5%, 10/2,184) (8). Finally, in June 2020, *E. cloacae* ST190 carrying *bla*_NDM-1_ gene located in a truncated insertion sequence IS*Aba125* on a IncC plasmid was reported in four patients (20). Additionally, one *E. coli* isolate and one *K. pneumoniae* isolate both carrying *bla*_NDM-1_ were also identified (20).

For Sri Lanka, a total of 15 studies addressed the presence of CRE in the country (Figure 3; Supplementary Table S2). In 2012, a four-month study detected 22 *K. pneumoniae* isolates belonging to ST14, ST147, ST380, carrying *bla*_OXA-181_ and *bla*_NDM-1_ genes in a hospital (21). In early 2013, one isolate of *K. pneumoniae* ST394 with *bla*_NDM-1_ on a IncHI plasmid, which included the insertion sequence IS*Aba125* upstream, was detected (22). Throughout the 2013 year, four *E. coli* isolates (resistance rate: 7.5%) and ten *K. pneumoniae* isolates (resistance rate: 40.0%) were identified in a tertiary hospital (23). In 2014, a national laboratory-based surveillance recorded a total of 149 CRE isolates (resistance rate: 9.0%) (24). During the first semester of 2015, a descriptive cross-sectional study reported the presence of *E. coli* (resistance rate: 4.9%) and *Klebsiella* spp. resistant to carbapenems (25). In addition, an eight-month study conducted in 2015 described carbapenemase-producing *K. pneumoniae* ST147 and ST437 with *bla*_OXA-181_ gene in ten patients. Three plasmids, CUHK_SL-A, CUHK_SL-B, and CUHK_SL-C, were identified to carry the gene, with the CUHK_SL-A plasmid harboring the insertion sequence IS*Ecp*, while the CUHK_SL-B plasmid did not have this insertion sequence (26). The CUHK_SL-C plasmid presented both IS*Ecp1* and a mobile gene (*mobC*) deletion (26). Between March and September 2015, *E. coli* and *K. pneumoniae* were also reported during a retrospective study in a hospital (27). In 2015–2016, three isolates of *K. pneumoniae* ST147 carrying the *bla*_OXA-181_ gene, two isolates of *K. pneumoniae* ST16 harboring *bla*_OXA-181_ and *bla*_OXA-232_ genes, one isolate of *Enterobacter hormaechei* subsp. *steigerwaltii* (*E. cloacae* complex) ST93 carrying *bla*_OXA-181_ were reported in patients presenting hospital-acquired urinary tract infections. All resistance genes were localized on the ColKP3 plasmid and flanked by the insertion sequence IS*Ecp1* (28). In a neonatal unit between October 2015 and January 2016, one carbapenem-resistant *Klebsiella* spp. isolate was detected (29). Additionally, between 2015 and 2016, the hospital reported 15 isolates of *E. coli* (resistance rate: 5.1%) carrying the *bla*_NDM-1_ gene, 24 isolates of *K. pneumoniae* (resistance rate: 36.9%) presenting *bla*_NDM-1_, *bla*_OXA-181_ and *bla*_OXA-232_ genes, three isolates of *Enterobacter* spp. (resistance rate: 11.5%) harboring *bla*_NDM-1_, *bla*_NDM-4_ and *bla*_OXA-181_, as well as four other Enterobacterales isolates (resistance rate: 10.5%) (30). In December 2016 and March 2017, one isolate of *E. coli* and one isolate of *K. pneumoniae* (resistance rate: 2.7%) resistant to carbapenem were retrieved (31). Between December 2017 and February 2018, a prospective cross-sectional study identified 57 CRE isolates in 57 patients (32). These isolates included *K. pneumoniae* carrying *bla*_KPC_, *bla*_OXA-48-*like*_, and both *bla*_NDM_ and *bla*_OXA-48-*like*_; *E. coli* carrying the *bla*_OXA-48-*like*_ gene, *C. freundii* carrying *bla*_OXA-48-*like*_ and *bla*_NDM_, *Providencia rettgeri* and *E. cloacae* both carrying *bla*_NDM_; and *Klebsiella aerogenes* harboring both *bla*_NDM_ and *bla*_OXA-48-*like*_ (32). In a separate study between August 2016 and January 2017, four carbapenem-resistant *K. pneumoniae* isolates were detected in four infants in a post-partum ward (33). Additionally, between 2018 and 2019, a nine-month descriptive cross-sectional study reported the presence of 37 CRE isolates (resistance rate: 41.1%) among patients with cancer in a hospital (34). Finally, in another study, 119 CRE isolates originating from 93 patients with cancer (rate of CRE carriers: 35.2%) were detected and found to carry *bla*_NDM_, *bla*_OXA-48_ and *bla*_KPC_ genes (35).

For Zanzibar, only one study reported the detection of a carbapenem-resistant *K. pneumoniae* isolate in a neonatal unit (resistant rate: 9.1%, 1/11; carrier rate: 0.2%, 1/469) (Figure 3; Supplementary Table S2) (36).

### 3.2 *Acinetobacter* spp. resistant to carbapenems

**Figure 4:**
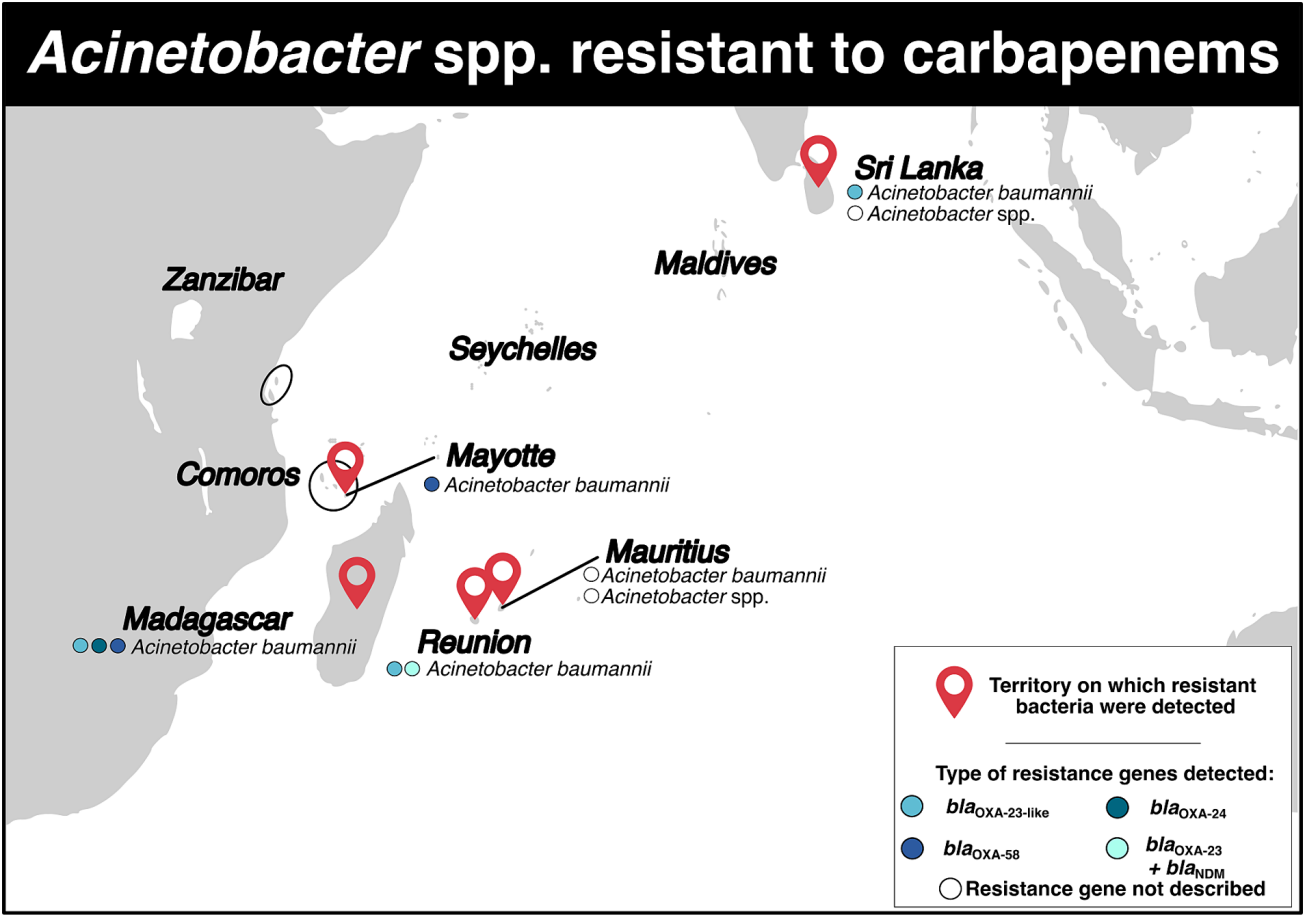
Distribution of carbapenem-resistant *Acinetobacter* spp. and genes associated with resistance mechanisms in the Southwest Indian Ocean Islands.

Carbapenem-resistant *Acinetobacter baumannii* (CRAB) was detected on five territories (Figure 4; Supplementary Tables S2, S3). Three studies were available for Madagascar. From September 2006 to March 2008, 50 isolates of CRAB (resistance rate: 44.7%) were identified in patients in intensive care and surgery wards (37). From September 2006 to March 2009, 53 CRAB isolates were identified in various Malagasy hospitals (resistance rate: 44.0%) (38). All isolates contained the *bla*_OXA-23_ gene carried by the insertion sequence IS*Aba1* (38). Finally, between 2008 and 2016, 15 CRAB isolates belonging to four STs (ST1, ST2, ST1195, ST1196) were detected, and the *bla*_OXA-23_, *bla*_OXA-24_ and *bla*_OXA-58_ genes were identified. The *bla*_OXA-23_ gene was located in Tn*2006* and Tn*2008* transposons on the bacterial chromosome, with the insertion sequence IS*Ab1* upstream (39). The *bla*_OXA-24_ was flanked by XerC/XerD recombination sites on the small designated pOXA-24_AB334 plasmid (39).

For Mauritius, three studies were included (Figure 4; Supplementary Table S2). In 2010 and 2014, the resistance rates for *Acinetobacter* spp. isolates were 68.0 and 74.0% respectively, indicating that a majority of the isolates were carbapenem-resistant in hospitalized patients (11, 12). Another retrospective study conducted between July 2015 and December 2016 identified 32 CRAB isolates, with a resistance rate of 86.5% (13).

On Mayotte Island, one study mentioned the detection of two CRAB isolates in May and August 2011 (Figure 4; Supplementary Table S2). These isolates belonged to ST23 and carried the *bla*_OXA-58_ gene on the bacterial chromosome, with the insertion sequence IS*Aba3* downstream (40). It was suggested that these isolates likely originated from the Comoros archipelago, specifically from Grande Comore and Mohéli islands (40).

On Reunion Island, a comparative study conducted between 1997 and 2005 revealed a decrease in carbapenem resistance for *A. baumannii* from 12.9 to 8.3% (41). Another study reported the presence of a CRAB isolate belonging to ST2 and carrying the *bla*_OXA-23-*like*_ gene in hospital (Figure 4; Supplementary Table S2) (42). In 2017, a woman who had previously traveled to Saudi Arabia was found to have a OXA-23 carbapenemase-producing *A. baumannii* (17). More recently, during an outbreak from April 2019 and June 2020, CRAB isolates were obtained from 13 patients. The isolates belonged to ST^Pas^1/ST^Ox^231 clonal complex and carried the *bla*_NDM-1_ and *bla*_OXA-23_ genes. The *bla*_NDM-1_ gene was located on the Tn*125* transposon, while the *bla*_OXA-23_ gene was located on the Tn*2006* transposon (Figure 4; Supplementary Table S2) (43). All the 13 isolates displayed resistance to colistin (43).

In Sri Lanka, three studies were included in the analysis (Figure 4; Supplementary Table S2). In a one-year study conducted in 2013, it was reported that 18 carbapenem-resistant *Acinetobacter* spp. were detected in in a tertiary care hospital with resistance rate of 87.5% (23). Another study from March to September 2015 found 30 carbapenem-resistant *Acinetobacter* spp. in an intensive care unit (27). These isolates were also found to be multidrug-resistant (27). More recently, 46 CRAB isolates carrying *bla*_OXA-23-*like*_ were isolated (44).

### 3.3 *Pseudomonas* spp. resistant to carbapenems

**Figure 5:**
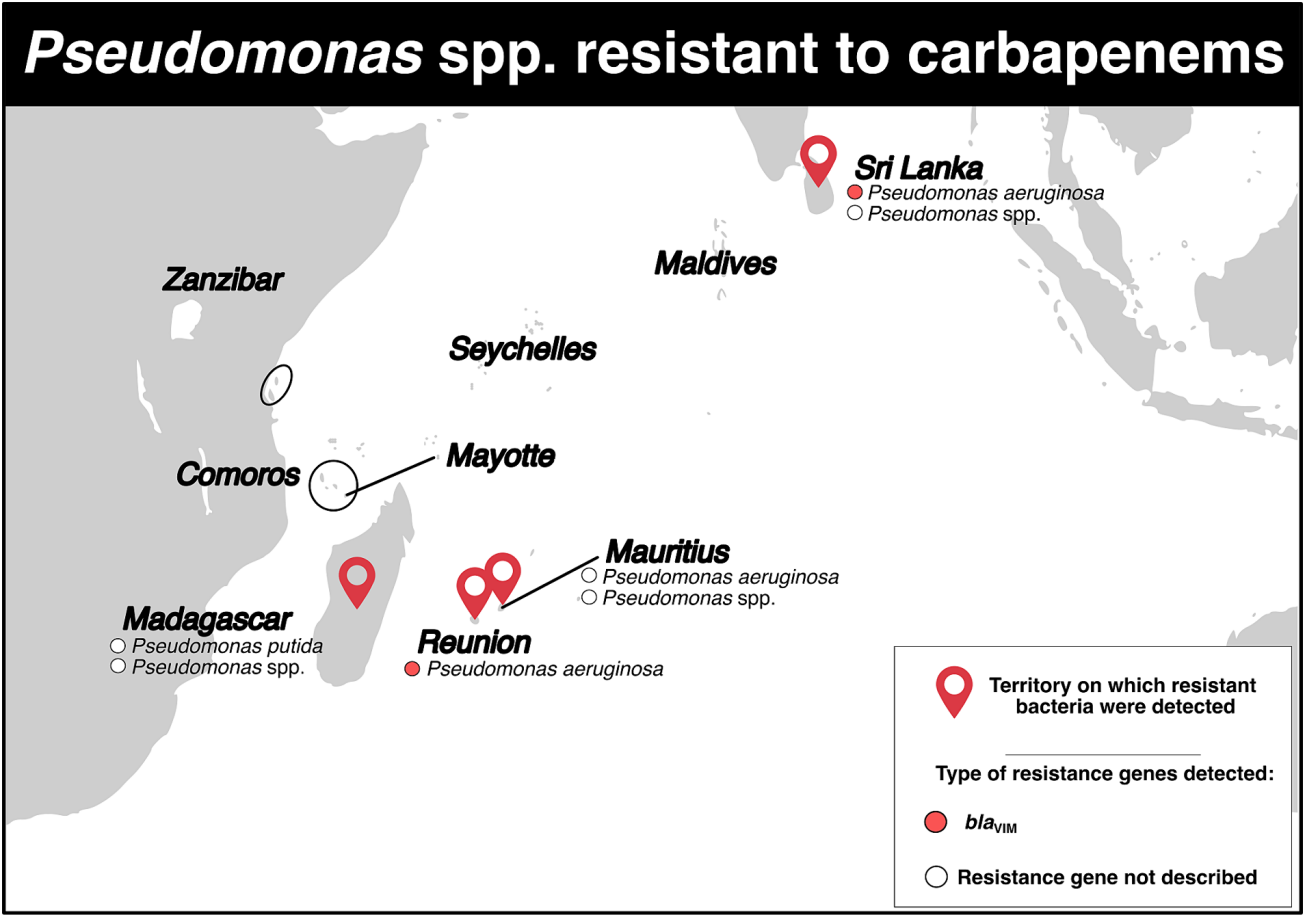
Distribution of carbapenem-resistant *Pseudomonas* spp. and genes associated with resistance mechanisms in the Southwest Indian Ocean Islands.

Carbapenem-resistant *Pseudomonas* spp. isolates were reported in four territories (Figure 5; Supplementary Tables S2, S3). In Madagascar, only two studies have described the detection of these bacteria. The first study, conducted between September 2006 and March 2008, reported a resistance rate of 1.9% for *Pseudomonas* spp. in an intensive care unit (37). The second study, published in 2015 and conducted in a hospital and community setting, reported the detection of three *Pseudomonas putida* isolates that were intermediate susceptible to carbapenems (resistance rate: 60%), but still susceptible to other antibiotics (7).

In Mauritius, a study in 2010 reported a 40.0% resistance rate to carbapenems in *Pseudomonas aeruginosa* (86 isolates) in a hospital setting (11). Similarly, another study in July 2014 reported a 27.0% resistance rate, also in a hospital (12). A retrospective study conducted between July 2015 and December 2016 reported a total of 16 *Pseudomonas* spp. isolates resistant to carbapenems (resistance rate: 80.0%) (Figure 5; Supplementary Table S2) (13).

Between 1997 and 2005, there was a stable resistance rate to carbapenems in Reunion Island. The rate of *P. aeruginosa* resistant to carbapenems ranged from 5.9 to 6.1% (41). From January 2010 to June 2012, three isolates of *P. aeruginosa* producing VIM-2 and one producing VIM-6 were identified (Figure 5; Supplementary Table S2) (45).

In Sri Lanka, in 2000, three carbapenem-resistant *P. aeruginosa* isolates belonging to ST235 and carrying the *bla*_VIM-2_ gene on their chromosome were detected (46). Between January and December 2013, a tertiary care hospital reported two isolates of *Pseudomonas* spp. displaying resistance to carbapenems, with a resistance rate of 10.0% (Figure 5; Supplementary Table S2) (23). During a retrospective study conducted in a Sri Lankan intensive care unit between March and September 2015, two *P. aeruginosa* isolates resistant to carbapenem were detected, with a resistance rate of 13.3% (Figure 5; Supplementary Table S2) (27).

### 3.4 Enterococci resistant to vancomycin

**Figure 6:**
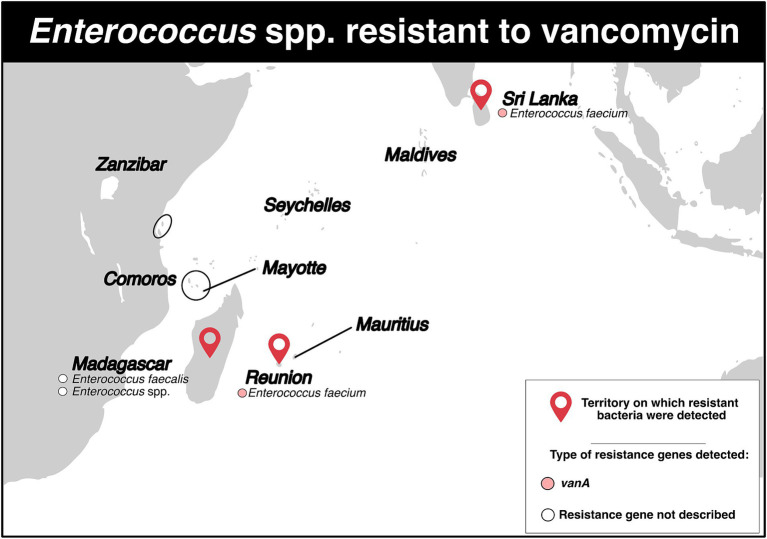
Distribution of vancomycin-resistant *Enterococcus* spp. and genes associated with resistance mechanisms in the Southwest Indian Ocean Islands.

VRE were reported in six studies across three territories (Figure 6; Supplementary Tables S2, S3). Two of the studies were performed in Madagascar. The first one, a cross-sectional survey conducted between 2006 and 2008 in surgery and intensive care wards, detected one resistant *Enterococcus* spp. isolate (resistance rate: 3.3%) (37). The second study, conducted between January 2011 and December 2013, detected one resistant *Enterococcus faecalis* isolate in the community population (resistance rate: 5.6%) (6).

On Reunion Island, three studies were identified (Figure 6; Supplementary Table S2). Between January 2015 and December 2017, one patient tested positive for resistant *Enterococcus faecium* (rate of VRE carriers: 0.05%) (8). In 2017, a patient repatriated from Mauritius tested positive for a resistant *E. faecium* that carried the *vanA* gene (18). Moreover, between January 2015 and December 2019, 16 resistant isolates of *E. faecium* harboring the *vanA* gene were detected. Half of these isolates were also resistant to linezolid. One isolate even exhibited simultaneous resistance to vancomycin, teicoplanin, linezolid and daptomycin. Among the 16 isolates, six (37.5%) showed a connection to a foreign country: two with India, two with Mauritius, one with Madagascar, and one with India/Saudi Arabia (47). Genotyping analyses identified five STs, with ST761 (*n* = 8), ST80 (*n* = 4) and ST5 (*n* = 2) being the most common.

Finally, in Sri Lanka, between January and March 2012, 11 *E. faecium* isolates were obtained from 11 patients (VRE carrier rate: 5.1%), and the *vanA* gene was detected in all isolates (Figure 6; Supplementary Table S2) (48).

## 4 Discussion

In this systematic review, we screened the literature to assess the current knowledge of critical- and high-priority resistant bacteria in several territories including Comoros, Madagascar, Maldives, Mauritius, Mayotte, Reunion Island, Seychelles, Sri Lanka, and Zanzibar. Similarly to Gay et al. in 2017, we encountered challenges due to the diverse study designs, resulting in uneven information across the available studies (4). However, our findings indicate that the targeted bacteria are actively circulating in the SWIO area. Gram-negative bacilli appeared to be most prevalent in the eastern islands of the region, particularly Mauritius and Sri Lanka, where resistance rates for some bacterial species can be alarmingly high (e.g., up to 87% of resistant *A. baumannii* in Mauritius and up to 100% in Sri Lanka). However, it is important to note that these findings may be affected by a bias, as studies were lacking for some territories. As far VRE, the limited number of studies and their distribution across different territories make it difficult to draw firm conclusions. Finally, cases of co-resistance have been recorded in the region, such as carbapenem-colistin in *A. baumannii* or vancomycin-teicoplanin-linezolid-daptomycin resistance in *E. faecium*, raising serious concerns about the availability of effective therapeutic alternatives for infections caused by this type of extensively-drug resistant bacteria.

With further details, CPE were the bacteria for which more data were available. They were relatively widespread and were detected in two-thirds of the investigated territories. Six resistance genes families were associated with resistance mechanisms; however, the *bla*_NDM_ and *bla*_OXA-48-like_ families were the most represented, supporting the trend observed worldwide (49–51). Interestingly, during our literature screening, emerging high-risk clones were detected. Specifically, *K. pneumoniae* ST307/ST147 or *E. coli* ST167/ST405/ST410 were reported on Reunion Island and/or Sri Lanka (19, 21, 26, 28). These clones are considered a significant threat to public health due to their propensity to harbor multiple-resistance genes, promoting their spread (particularly in regions where antibiotic use is poorly controlled); and because they can be involved in serious infections, as limited therapeutic options exist to treat infected patients (52, 53). Their presence on these islands might likely originate from importation from territories on which they already circulate. For instance, *K. pneumonia* ST147 might have been imported from India, where it has been previously reported (52), and for which extensive human traveling exchanges exist between the Indian subcontinent and the SWIO region. Carbapenem-resistant *Acinetobacter* spp. and *Pseudomonas* spp. were detected in half of the investigated territories. However, compared to CPE, the number of studies and available information were less extensive. *A. baumannii* was the dominant species, and resistance mechanisms were only associated with *bla*_NDM_ and *bla*_OXA_ genes. For *Pseudomonas* spp., the main represented species was *P. aeruginosa* and *bla*_VIM_ was the main gene involved in the resistance mechanisms. Finally, the bacteria that had the least number of available studies was *Enterococcus* spp. resistant to vancomycin. These bacteria were reported only in one third of the targeted territories. Both *E. faecalis* and *E. faecium* were identified and only the *vanA* gene was found to be associated with resistance to glycopeptides (47, 48). The contrasting level of information found between the four bacterial groups may be due to a bias in investigation and should be interpreted with caution when assessing the epidemiology of these critical and high-priority pathogens. It is possible that CPE has been the main focus for both scientific and medical communities in recent years, which may explain why there are more studies investigating their circulation compared to the other groups. However, these discrepancies might also originate from the socio-economic context of the region. The selected islands belong to eight countries with highly disparate gross domestic product *per capita* and healthcare systems. Health policies and resources allocated to investigate AMR, particularly through antimicrobial susceptibility testing, are not equal across these territories (Supplementary Table S2). For instance, bacterial identification involving colorimetric/biochemical methodologies might be less precise than new approaches such as Matrix-Assisted Laser Desorption Ionization-Time of Flight Mass Spectrometry (54). Similarly, the use of traditional molecular biology test (PCRs) and limited access to sequencing analysis capabilities hinder performing Next Generation Sequencing for resistome/bacterial genotyping (55) may explain the scarcity of data regarding the localization of resistance genes. Variations in the number of available studies across the nine investigated territories could also be attributed to the socio-economic context. Additionally, in regions with limited resources, the use of broad-spectrum antibiotic molecules like third generation cephalosporins or carbapenems as probabilistic treatments may contribute to the selection and proliferation of these resistant isolates. More globally, these socio-economic disparities and contact with highly endemic regions could also drive the spread of AMR through population movements, such as tourism or medical evacuation, as observed in many published examples (18, 19, 40). However, collaborative efforts with the IOC provide opportunities for multicenter studies to overcome recruitment biases. It is worth noting that veterinary surveillance targeting these critical and high priority pathogens is scarce, and that no “One Health” study has looked at the cross-compartmental spread of these pathogens in this geographical area, indicating a need for improvement that should be highlighted.

In conclusion, our review highlights a growing interest in studying AMR in the SWIO region. The identification of critical and high priority pathogens emphasizes the alarming progression of this global silent pandemic, even in insular ecosystems, and provides an overview of the regional epidemiology. Nevertheless, the available information is still lacking consistency among these territories. Furthermore, there is shortage of research on resistance mechanisms and genotyping analyses. It is, therefore, necessary to enhance the diagnostic capabilities of laboratories to collect more comprehensive data in the future. Now more than ever, it is crucial to set up a regional surveillance network to prevent the spread of these pathogens. This should be done alongside implementing strict and uniform infection control measures, as well as effective antibiotics stewardship.

## Author contributions

AH: Data curation, Formal analysis, Writing – original draft, Writing – review & editing. PM: Writing – review & editing. GM: Writing – review & editing.

## References

[1] Murray CJL Ikuta KS Sharara F Swetschinski L Robles Aguilar G Gray A. Global burden of bacterial antimicrobial resistance in 2019: a systematic analysis. Lancet. (2022) 399:629–55. doi: 10.1016/S0140-6736(21)02724-0, PMID: 35065702 PMC8841637

[2] World Health Organization. World Health Organization publishes list of bacteria for which new antibiotics are urgently needed. (2017) Available at: https://www.who.int/news/item/27-02-2017-who-publishes-list-of-bacteria-for-which-new-antibiotics-are-urgently-needed [Accessed August 18, 2023]

[3] COI. Rapport Annuel 2015. (2015) Available at: http://commissionoceanindien.org/fileadmin/resources/SG/Rapportannuel2015.pdf (Accessed September 03, 2023)

[4] Gay N Belmonte O Collard J-M Halifa M Issack MI Mindjae S. Review of antibiotic resistance in the Indian Ocean commission: a human and animal health issue. Front. Public Health. (2017) 5:162. doi: 10.3389/fpubh.2017.00162, PMID: 28730149 PMC5498788

[5] Moher D Liberati A Tetzlaff J Altman DG PRISMA Group. Preferred reporting items for systematic reviews and meta-analyses: the PRISMA statement. PLoS Med. (2009) 6:e1000097. doi: 10.1371/journal.pmed.1000097, PMID: 19621072 PMC2707599

[6] Rasamiravaka T Shaista Sheila HSL Rakotomavojaona T Rakoto-Alson AO Rasamindrakotroka A. Changing profile and increasing antimicrobial resistance of uropathogenic bacteria in Madagascar. Med Mal Infect. (2015) 45:173–6. doi: 10.1016/j.medmal.2015.03.006, PMID: 25866374

[7] Micheel V Hogan B Rakotoarivelo RA Rakotozandrindrainy R Razafimanatsoa F Razafindrabe T. Identification of nasal colonization with β-lactamase-producing Enterobacteriaceae in patients, health care workers and students in Madagascar. Eur J Microbiol Immunol (Bp). (2015) 5:116–25. doi: 10.1556/EUJMI-D-15-00001, PMID: 25908994 PMC4403799

[8] Gay N Lugagne N Miltgen G Belmonte O Cardinale E. Reunion Island, a sentinel territory for antimicrobial-resistant bacteria surveillance in the South-Western Indian Ocean: a retrospective survey using hospitalized patient screening, 2015–2017. BMC Public Health. (2020) 20:1488. doi: 10.1186/s12889-020-09591-8 33004028 PMC7528459

[9] Milenkov M Rasoanandrasana S Rahajamanana LV Rakotomalala RS Razafindrakoto CA Rafalimanana C. Prevalence, risk factors, and genetic characterization of extended-Spectrum Beta-lactamase *Escherichia coli* isolated from healthy pregnant women in Madagascar. Front Microbiol. (2021) 12:786146. doi: 10.3389/fmicb.2021.786146, PMID: 35003019 PMC8740230

[10] Poirel L Lascols C Bernabeu S Nordmann P. NDM-1-producing *Klebsiella pneumoniae* in Mauritius. Antimicrob Agents Chemother. (2012) 56:598–9. doi: 10.1128/AAC.05639-11, PMID: 22006002 PMC3256058

[11] Issack M Manraj S. Antibiotic susceptibility of bacteria isolated from hospitalized patients in Mauritius. (2011) Available at: https://f1000research.com/posters/1089854 [Accessed September 12, 2023]

[12] Issack M. Antibiotic resistance among hospitalized patients in Mauritius in 2014. Int J Infect Dis. (2016) 45:94. doi: 10.1016/j.ijid.2016.02.250

[13] Nuckchady DC Boolaky SH. The Prevalence of Multi-Drug Resistant Organisms and Their Outcomes in an ICU in Mauritius: An Observational Study Asian. J Med Health. (2020) 18:71–8. doi: 10.9734/ajmah/2020/v18i1130270

[14] Miltgen G Bonnin RA Avril C Benoit-Cattin T Martak D Leclaire A. Outbreak of IMI-1 carbapenemase-producing colistin-resistant *Enterobacter cloacae* on the French island of Mayotte (Indian Ocean). Int J Antimicrob Agents. (2018) 52:416–20. doi: 10.1016/j.ijantimicag.2018.05.015, PMID: 29807164

[15] Cabanes F Lemant J Picot S Simac C Cousty J Jalin L. Emergence of *Klebsiella pneumoniae* and *Salmonella* Metallo-Beta-lactamase (NDM-1) producers on Reunion Island. J Clin Microbiol. (2012) 50:3812–2. doi: 10.1128/JCM.01029-12, PMID: 22972814 PMC3486249

[16] Holman AM Allyn J Miltgen G Lugagne N Traversier N Picot S. Surveillance of carbapenemase-producing Enterobacteriaceae in the Indian Ocean region between January 2010 and December 2015. Med Mal Infect. (2017) 47:333–9. doi: 10.1016/j.medmal.2017.04.007, PMID: 28602387

[17] Leroy AG Naze F Dortet L Naas T Jaubert J. Plasmid-mediated colistin resistance gene *mcr-1* in a clinical *Escherichia coli* isolate in the Indian Ocean commission. Med Mal Infect. (2018) 48:426–8. doi: 10.1016/j.medmal.2018.04.388, PMID: 29753527

[18] Allyn J Coolen-Allou N de Parseval B Galas T Belmonte O Allou N. Medical evacuation from abroad of critically ill patients: a case report and ethical issues. Medicine (Baltimore). (2018) 97:e12516. doi: 10.1097/MD.0000000000012516, PMID: 30235768 PMC6160182

[19] Miltgen G Cholley P Martak D Thouverez M Seraphin P Leclaire A. Carbapenemase-producing Enterobacteriaceae circulating in the Reunion Island, a French territory in the Southwest Indian Ocean. Antimicrob Resist Infect Control. (2020) 9:36. doi: 10.1186/s13756-020-0703-3, PMID: 32075697 PMC7031992

[20] Miltgen G Garrigos T Cholley P Deleume M Allou N Allyn J. Nosocomial cluster of carbapenemase-producing *Enterobacter cloacae* in an intensive care unit dedicated COVID-19. Antimicrob Resist Infect Control. (2021) 10:151. doi: 10.1186/s13756-021-01022-6, PMID: 34674756 PMC8529563

[21] Hall JM Corea E Sanjeewani HDA Inglis TJJ. Molecular mechanisms of β-lactam resistance in carbapenemase-producing *Klebsiella pneumoniae* from Sri Lanka. J Med Microbiol. (2014) 63:1087–92. doi: 10.1099/jmm.0.076760-0, PMID: 24855071

[22] Dortet L Brechard L Grenet K Nguessan M-S Nordmann P. Sri Lanka, another country from the Indian subcontinent with NDM-1-producing Enterobacteriaceae. J Antimicrob Chemother. (2013) 68:2172–3. doi: 10.1093/jac/dkt145, PMID: 23620468

[23] Jayatilleke K. Increasing antibiotic resistance in a tertiary care hospital in Sri Lanka. Sri Lankan J Infect Dis. (2014) 4:108. doi: 10.4038/sljid.v4i2.6851

[24] Jayatilleke S Patabendige G Dassanayake M Karunaratne G Perera J Perera R. Analysis of urine culture isolates from seven laboratories of Sri Lanka: national laboratory based surveillance of Sri Lanka College of Microbiologists in 2014. Sri Lankan J Infect Dis. (2016) 6:17. doi: 10.4038/sljid.v6i1.8105

[25] Fernando MMPSC Luke WANV Miththinda JKND Wickramasinghe RDSS Sebastiampillai BS Gunathilake MPML. Extended spectrum beta lactamase producing organisms causing urinary tract infections in Sri Lanka and their antibiotic susceptibility pattern—a hospital based cross sectional study. BMC Infect Dis. (2017) 17:138. doi: 10.1186/s12879-017-2250-y, PMID: 28187754 PMC5303299

[26] Zhu C Liyanapathirana V Li C Pinto V Hui M Lo N. Characterizing mobilized virulence factors and multidrug resistance genes in carbapenemase-producing *Klebsiella pneumoniae* in a Sri Lankan hospital. Front Microbiol. (2018) 9:2044. doi: 10.3389/fmicb.2018.02044, PMID: 30233529 PMC6127249

[27] Tissera K Liyanapathirana V Dissanayake N Pinto V Ekanayake A Tennakoon M. Spread of resistant gram negatives in a Sri Lankan intensive care unit. BMC Infect Dis. (2017) 17:490. doi: 10.1186/s12879-017-2590-7, PMID: 28697755 PMC5506608

[28] Perera V de Silva S Jayatilleke K de Silva N Aydin A Enne V. Antimicrobial resistance genes, virulence genes, and associated mobile genetic elements of eight multidrug-resistant Enterobacterales isolated from hospital-acquired urinary tract infections in Sri Lanka. Microb Drug Resist. (2022) 28:882–92. doi: 10.1089/mdr.2022.0003, PMID: 35972764

[29] Nanayakkara D Liyanapathirana V Kandauda C Gihan C Ekanayake A Adasooriya D. Maternal vaginal colonization with selected potential pathogens of neonatal sepsis in the era of antimicrobial resistance, a single center experience from Sri Lanka. BMC Infect Dis. (2018) 18:351. doi: 10.1186/s12879-018-3262-y, PMID: 30055584 PMC6064104

[30] Perera PDVM Gamage S De Silva HSM Jayatilleke SK de Silva N Aydin A. Phenotypic and genotypic distribution of ESBL, AmpC β-lactamase and carbapenemase-producing Enterobacteriaceae in community-acquired and hospital-acquired urinary tract infections in Sri Lanka. J Glob Antimicrob Resist. (2022) 30:115–22. doi: 10.1016/j.jgar.2022.05.024, PMID: 35667644

[31] Priyadharshana U Piyasiri LB Wijesinghe C. Prevalence, antibiotic sensitivity pattern and genetic analysis of extended spectrum beta lactamase producing *Escherichia coli* and *Klebsiella* spp. among patients with community acquired urinary tract infection in Galle district, Sri Lanka. Ceylon Med J. (2019) 64:140–5. doi: 10.4038/cmj.v64i4.8990, PMID: 32120467

[32] Kumudunie WGM Wijesooriya LI Namalie KD Sunil-Chandra NP Wijayasinghe YS. Epidemiology of multidrug-resistant Enterobacteriaceae in Sri Lanka: first evidence of *bla*_KPC_ harboring *Klebsiella pneumoniae*. J Infect Public Health. (2020) 13:1330–5. doi: 10.1016/j.jiph.2020.04.010, PMID: 32439355

[33] Meredith HR Kularatna S Nagaro K Nagahawatte A Bodinayake C Kurukulasooriya R. Colonization with multidrug-resistant Enterobacteriaceae among infants: an observational study in southern Sri Lanka. Antimicrob Resist Infect Control. (2021) 10:72. doi: 10.1186/s13756-021-00938-3, PMID: 33931120 PMC8086278

[34] Chathuranga G Dissanayake T Fernando N Wanigatunge C. Appropriateness of the empirical antibiotics prescribed and their concordance with national guidelines for three selected infections among cancer patients in a tertiary care center in Sri Lanka. Int J Microbiol. (2021) 2021:1–7. doi: 10.1155/2021/7572215, PMID: 34621317 PMC8492258

[35] Suranadee YWS Dissanayake Y Dissanayake BMBT Jyalatharachchi HR Gamage S Gunasekara SP. P31 gut colonization of carbapenem-resistant Enterobacteriaceae among patients with haematological malignancies in National Cancer Institute, Sri Lanka. JAC Antimicrob Resist. (2022) 4:dlac004.030. doi: 10.1093/jacamr/dlac004.030

[36] Onken A Said AK Jørstad M Jenum PA Blomberg B. Prevalence and antimicrobial resistance of microbes causing bloodstream infections in Unguja. Zanzibar PLoS One. (2015) 10:e0145632. doi: 10.1371/journal.pone.0145632, PMID: 26700032 PMC4689456

[37] Randrianirina F Vaillant L Ramarokoto CE Rakotoarijaona A Andriamanarivo ML Razafimahandry HC. Antimicrobial resistance in pathogens causing nosocomial infections in surgery and intensive care units of two hospitals in Antananarivo. Madagascar J Infect Dev Ctries. (2009) 4:074–82. doi: 10.3855/jidc.454 20212337

[38] Andriamanantena TS Ratsima E Rakotonirina HC Randrianirina F Ramparany L Carod J-F. Dissemination of multidrug resistant *Acinetobacter baumannii* in various hospitals of Antananarivo Madagascar. Ann Clin Microbiol Antimicrob. (2010) 9:17. doi: 10.1186/1476-0711-9-17, PMID: 20591154 PMC2910008

[39] Simo Tchuinte PL Rabenandrasana MAN Kowalewicz C Andrianoelina VH Rakotondrasoa A Andrianirina ZZ. Phenotypic and molecular characterizations of carbapenem-resistant *Acinetobacter baumannii* strains isolated in Madagascar. Antimicrob Resist Infect Control. (2019) 8:31. doi: 10.1186/s13756-019-0491-9, PMID: 30792853 PMC6371490

[40] Bonnin RA Poirel L Benoit-Cattin T Nordmann P. Ceftazidime-susceptible and imipenem-non-susceptible OXA-58-producing *Acinetobacter baumannii* from the Comoros archipelago. Int J Antimicrob Agents. (2013) 41:297–8. doi: 10.1016/j.ijantimicag.2012.11.002, PMID: 23313400

[41] Picot S Rakotomalala RS Farny K Simac C Michault A. Évolution de la résistance aux antibiotiques de 1997 à 2005 à La Réunion. Med Mal Infect. (2010) 40:617–24. doi: 10.1016/j.medmal.2010.04.001, PMID: 20570074

[42] Pailhoriès H Belmonte O Kempf M Lemarié C Cuziat J Quinqueneau C. Diversity of *Acinetobacter baumannii* strains isolated in humans, companion animals, and the environment in Reunion Island: an exploratory study. Int J Infect Dis. (2015) 37:64–9. doi: 10.1016/j.ijid.2015.05.012, PMID: 26093214

[43] Miltgen G Bour M Allyn J Allou N Vedani T Vuillemenot J-B. Molecular and epidemiological investigation of a colistin-resistant OXA-23-/NDM-1-producing *Acinetobacter baumannii* outbreak in the Southwest Indian Ocean area. Int J Antimicrob Agents. (2021) 58:106402. doi: 10.1016/j.ijantimicag.2021.106402 34293453

[44] Alles MFJ. Molecular Detection of Selected Genetic Determinants of carbapenem resistance among invasive isolates of *Acinetobacter baumannii* recovered from selected tertiary care units in Colombo District, Sri Lanka. (2021). Available at: http://librepository.pgim.cmb.ac.lk/handle/1/4247 [Accessed September 23, 2023]

[45] Jeannot K Belmonte O Fournier D Robert-Nicoud R Müller E Plésiat P. Epidémiologie des β-lactamases à spectre élargi (BLSE) et des carbapénèmases chez *Pseudomonas aeruginosa* sur l’île de la Réunion. Paris: Réunion Interdisciplinaire de Chimiothérapie Anti-Infectieuse (2012).

[46] Kim MJ Bae IK Jeong SH Kim SH Song JH Choi JY. Dissemination of metallo-β-lactamase-producing *Pseudomonas aeruginosa* of sequence type 235 in Asian countries. J Antimicrob Chemother. (2013) 68:2820–4. doi: 10.1093/jac/dkt269, PMID: 23843299

[47] Kamus L Auger G Gambarotto K Houivet J Ramiandrisoa M Picot S. Investigation of a *vanA* linezolid- and vancomycin-resistant *Enterococcus faecium* outbreak in the Southwest Indian Ocean (Reunion Island). Int J Antimicrob Agents. (2022) 60:106686. doi: 10.1016/j.ijantimicag.2022.106686, PMID: 36503708

[48] Kannangara C Chandrasiri P Corea EM. Vancomycin resistant enterococcal (VRE) colonization among patients treated in intensive care units at the National Hospital of Sri Lanka, and determination of genotype/s responsible for resistance. Ceylon Med J. (2018) 63:154. doi: 10.4038/cmj.v63i4.8766 30669209

[49] Wu W Feng Y Tang G Qiao F McNally A Zong Z. NDM metallo-β-lactamases and their bacterial producers in health care settings. Clin Microbiol Rev. (2019) 32:e00115–8. doi: 10.1128/CMR.00115-18, PMID: 30700432 PMC6431124

[50] Jean S-S Harnod D Hsueh P-R. Global threat of carbapenem-resistant gram-negative bacteria. Front Cell Infect Microbiol. (2022) 12:823684. doi: 10.3389/fcimb.2022.823684, PMID: 35372099 PMC8965008

[51] Pitout JDD Peirano G Kock MM Strydom K-A Matsumura Y. The global ascendency of OXA-48-type carbapenemases. Clin Microbiol Rev. (2019) 33:e00102–19. doi: 10.1128/CMR.00102-19, PMID: 31722889 PMC6860007

[52] Peirano G Chen L Kreiswirth BN Pitout JDD. Emerging antimicrobial-resistant high-risk *Klebsiella pneumoniae* clones ST307 and ST147. Antimicrob Agents Chemother. (2020) 64:e01148–20. doi: 10.1128/AAC.01148-20 32747358 PMC7508593

[53] Garcia-Fernandez A Villa L Bibbolino G Bressan A Trancassini M Pietropaolo V. Novel insights and features of the NDM-5-producing *Escherichia coli* sequence type 167 high-risk clone. mSphere. (2020) 5:e00269–20. doi: 10.1128/mSphere.00269-20, PMID: 32350092 PMC7193042

[54] Florio W Baldeschi L Rizzato C Tavanti A Ghelardi E Lupetti A. Detection of antibiotic-resistance by MALDI-TOF mass spectrometry: an expanding area. Front Cell Infect Microbiol. (2020) 10:572909. doi: 10.3389/fcimb.2020.572909, PMID: 33262954 PMC7686347

[55] Ben Khedher M Ghedira K Rolain J-M Ruimy R Croce O. Application and challenge of 3rd generation sequencing for clinical bacterial studies. Int J Mol Sci. (2022) 23:1395. doi: 10.3390/ijms23031395, PMID: 35163319 PMC8835973

