# Corrigendum: Comprehensive analysis of antimicrobial resistance in the Southwest Indian Ocean: focus on WHO critical and high priority pathogens

**DOI:** 10.3389/fpubh.2024.1429799

**Published:** 2024-06-04

**Authors:** Axel O. G. Hoarau, Patrick Mavingui, Guillaume Miltgen

**Affiliations:** ^1^Université de La Réunion, Unité Mixte de Recherche Processus Infectieux en Milieu Insulaire Tropical (UMR PIMIT), INSERM 1187, CNRS 9192, IRD 249, Sainte-Clotilde, La Réunion, France; ^2^Laboratoire de Bactériologie, CHU Félix Guyon, Saint-Denis, La Réunion, France; ^3^Centre Régional en Antibiothérapie (CRAtb) de La Réunion, Saint-Pierre, La Réunion, France

**Keywords:** antimicrobial resistance, Enterobacterales, *Pseudomonas* spp., *Acinetobacter* spp., *Enterococcus* spp., carbapenem resistance, vancomycin resistance, Indian Ocean

In the published article, there was an error in affiliation 1. Instead of “Université de La Réunion, Processus Infectieux en Milieu Insulaire Tropical, Sainte-Clotilde, La Réunion, France”, it should be “Université de La Réunion, Unité Mixte de Recherche Processus Infectieux en Milieu Insulaire Tropical (UMR PIMIT), INSERM 1187, CNRS 9192, IRD 249, Sainte-Clotilde, La Réunion, France”.

The authors apologize for this error and state that this does not change the scientific conclusions of the article in any way. The original article has been updated.

